# Performance Evaluation of *In Vitro* Screening and Diagnostic Kits for Hepatitis C Virus Infection

**DOI:** 10.3389/fcimb.2021.793472

**Published:** 2022-02-03

**Authors:** Asako Murayama, Haruka Momose, Norie Yamada, Keiji Matsubayashi, Masamichi Muramatsu, Isao Hamaguchi, Takanobu Kato

**Affiliations:** ^1^ Department of Virology II, National Institute of Infectious Diseases, Tokyo, Japan; ^2^ Department of Safety Research on Blood and Biological Products, National Institute of Infectious Diseases, Tokyo, Japan; ^3^ Central Blood Institute, Blood Service Headquarters, Japanese Red Cross Society, Tokyo, Japan

**Keywords:** HCV, diagnostics, antibody, antigen, RNA

## Abstract

**Aim:**

A reliable kit with high sensitivity and specificity is indispensable for diagnosing hepatitis C virus (HCV) infection. Detection kits for anti-HCV antibodies (anti-HCV) are used for screening, and quantification kits for HCV RNA and HCV antigen (Ag) are used for the definite diagnosis of HCV infection or the evaluation of the pathological condition of and therapeutic effects in patients with chronic hepatitis C. Several kits are currently available for these purposes and are provided for clinical use in Japan. In this study, we aimed to evaluate the performance of these kits.

**Methods:**

We used International Standards for HCV RNA and HCV Ag and a regional reference panel to evaluate the performance of thirteen anti-HCV, five HCV RNA, and two HCV Ag kits.

**Results:**

All specimens in the regional reference panel were diagnosed correctly by all anti-HCV kits, although the distributions of the quantified values varied, and the ratios of titer classification were not identical across kits. All HCV RNA kits quantified the International Standard with minimum deviation and diagnosed the specimens of the reference panel correctly. The quantified values of the International Standard by two HCV Ag kits were inconsistent. HCV Ag titers of some specimens were underestimated owing to the amino acid polymorphisms in comparison with HCV RNA titers.

**Conclusions:**

The evaluation with International Standards and the regional reference panel was useful for assessing the quality of screening and diagnostic kits for HCV infection, and such quality control is essential for the clinical usage of these kits.

## Introduction

Hepatitis C virus (HCV) is a human pathogen and major cause of chronic hepatitis. Approximately 71 million people globally have chronic hepatitis C infection (WHO, 2017). In most cases of infection, HCV causes chronic hepatitis and cirrhosis and leads to the development of hepatocellular carcinoma. Historically, interferon-based therapies have been used to eradicate HCV infection. However, these therapies often have unsatisfactory levels of efficacy, and chronic hepatitis C has been recognized to be a refractory disease (Feld and Hoofnagle, 2005; Pawlotsky, 2006; Feld and Ward, 2021). Recently, novel anti-HCV therapies with direct-acting antivirals have been established, and the number of patients who respond to treatment has increased (Pawlotsky, 2013; Feld, 2014; Welzel and Zeuzem, 2014; Tojima et al., 2020; Bhattacharjee et al., 2021; Stanciu et al., 2021). Therefore, chronic hepatitis C is now considered to be a curable disease, and it has been recommended to proceed with the detection of untreated or undiagnosed individuals with chronic HCV infection.

Currently, there are many *in vitro* screening and diagnostic kits for HCV infection available in Japan and other countries. These kits are inspected by the national regulatory authority in each country to verify their qualities before being released to the market. In Japan, when releasing new kits, the regulatory authority requires the same or better performance than preexisting kits on the market; the regression coefficient (slope of the correlation equation) of quantified values between new and preexisting kits should be between 0.9 and 1.1, and the coefficient of determination (R^2^) of these kits should be over 0.9. On the other hand, postmarketing surveillance is also considered to be important in maintaining the performance of these kits. Several kits are known to be affected by HCV genotypes and polymorphisms of nucleotides or amino acids, and predominant genotypes or strains may have regional specificity and change over time (Saeed et al., 2009; Murayama et al., 2012; Momose et al., 2018). To assess the performance of *in vitro* screening and diagnostic kits for HCV infection, International Standards for these kits and a regional reference panel are suitable. International Standards are approved by the World Health Organization (WHO) as a master calibrator of the international unit (IU), and a regional reference panel is built up by gathering the samples that are endemic in each region.

In this study, using International Standards for HCV RNA and HCV Ag, as well as a recently established regional reference panel, we evaluated a number of *in vitro* screening and diagnostic kits, including 13 anti-HCV detection, 5 HCV RNA quantification, and 2 HCV Ag quantification kits.

## Methods

### International Standards of HCV RNA and HCV Ag

International Standards are approved by the WHO for calibration of IUs. The sixth International Standard for HCV RNA genotype (GT) 1a (IS-RNA; NIBSC code: 18/184) and the first International Standard for HCV core antigen GT-1a (IS-Ag; PEI code: 129096/12) were obtained from the National Institute of Biological Standards and Control (NIBSC, UK) and Paul-Ehrlich-Institut (PEI, Germany), respectively. These International Standards were stored at -80°C until use.

### Establishment of Regional Reference Panel

The regional HCV reference panel was established with 70 HCV-negative (CN1–CN70) and 80 HCV-positive (C1–C80) plasma specimens that were provided by the Japanese Red Cross Blood Centers. These specimens were collected in 2014 and 2015 in Japan and were determined to be ineligible for transfusion. The HCV genomes in samples of the positive reference panel were amplified by nested PCR, and genotypes were determined as reported previously (Furui et al., 2011). The positive reference panel included HCV strains of GT-1b (28; 35.0%), GT-2a (26; 32.5%), and GT-2b (26; 32.5%), which are in agreement with the predominant genotypes in Japan. These specimens were aliquoted in 1.0-mL volumes into 1.5-mL screw-cap tubes after centrifugation to exclude agglutinates or clots and then stored at −80°C until use.

### Evaluation of HCV Detection Kits

In this study, 13 anti-HCV detection kits were enrolled: ARCHITECT Anti-HCV (ARCHITECT-Ab; Abbott Japan, Tokyo, Japan) (Yoo et al., 2015; Shepherd et al., 2020; Nam et al., 2021), Alinity i Anti-HCV (Alinity-Ab; Abbott Japan) (Shepherd et al., 2020; Nam et al., 2021), Lumipulse HCV (Lumipulse-Ab; Fujirebio, Tokyo, Japan), Lumipulse Presto HCV (Lumipulse Presto-Ab; Fujirebio), Lumipulse II Ortho HCV (Ortho-Ab; Fujirebio), Lumipulse Presto Ortho HCV (Ortho Presto-Ab; Fujirebio), Elecsys Anti-HCV II (Elecsys-Ab; Roche Diagnostics, Tokyo, Japan) (Yoo et al., 2015; Feng et al., 2016; Xu et al., 2019), Accuraseed HCV [II], (Accuraseed-Ab; Sanyo Chemical Industries, Kyoto, Japan), ADVIA Centaur anti-HCV assay (Centaur-Ab; Siemens Healthcare Diagnostics, Tokyo, Japan) (Dwyer, 2004; Yoo et al., 2015), HISCL Anti-HCV Assay Kit (HISCL-Ab; Sysmex, Kobe, Japan) (Feng et al., 2016), HISCL Anti-HCV II Assay Kit (HISCL II-Ab; Sysmex) (Xu et al., 2019), CL AIA-PACK HCVAb (CL AIA-Ab; TOSOH, Tokyo, Japan), and E Test ‘TOSOH’ II HCVAb (AIA-Ab; TOSOH). Of these anti-HCV detection kits, Lumipulse-Ab, Lumipulse Presto-Ab, Ortho-Ab, Ortho Presto-Ab, Accuraseed-Ab, HISCL-Ab, and CL AIA-Ab provide classification titers.

Five HCV RNA quantification kits were enrolled: COBAS AmpliPrep/COBAS TaqMan HCV test version 2.0 (CAP/CTM-RNA2; Roche Diagnostics) (Zitzer et al., 2013; Momose et al., 2018; Yao et al., 2018), cobas HCV (cobas-RNA; Roche Diagnostics) (Yao et al., 2018), Abbott RealTime HCV assay (ART-RNA; Abbott Japan), Alinity m HCV assay (Alinity-RNA; Abbott Japan) (Chevaliez et al., 2020) and Aptima HCV (Aptima-RNA; Hologic Japan, Tokyo, Japan) (Schalasta et al., 2016; Chevaliez et al., 2017; Schonning et al., 2017; Worlock et al., 2017).

Two HCV Ag quantification kits were enrolled: ARCHITECT HCV Ag (ARCHITECT-Ag; Abbott Japan) (Leary et al., 2006; Morota et al., 2009) and Lumipulse Ortho HCV Ag (Lumipulse-Ag; Fujirebio). All assays were performed by the respective manufacturers at their research laboratories.

### Sequencing of HCV in the Regional Reference Panel

Viral RNA was extracted from 140 μL of each plasma sample using the QIAamp viral RNA kit (Qiagen, Valencia, CA, USA), and cDNA synthesis was conducted using SuperScript IV Reverse Transcriptase (Thermo Fisher Scientific, Waltham, MS). PCR was performed with a set of primers covering from the 5′ untranslated region (UTR) to the Envelope 1 region (1st round PCR: 44S-IH: 5′-CTGTGAGGAACTACTGTCTT-3′ and 1323R-IH: 5′-GGCGACCAGTTCATCATCAT-3′; 2nd round PCR: CS-17ssp: 5’-CCGGGAGAGCCATAGTGGTCTGCG-3’ and 1323R-IH). The amplified products were directly sequenced. The determined sequences of the core region were submitted to DDBJ/EMBL/GenBank and will appear with the following accession numbers: LC658661- LC658679.

### Statistical Analysis

Correlations of the quantitative data were assessed by linear regression analyses, and R^2^ values were calculated. P values of <0.05 were considered to indicate statistical significance. Analysis was performed using Prism 7 software (GraphPad Software, Inc., La Jolla, CA).

## Results

### Evaluation of Anti-HCV Detection Kits

**Figure 1:**
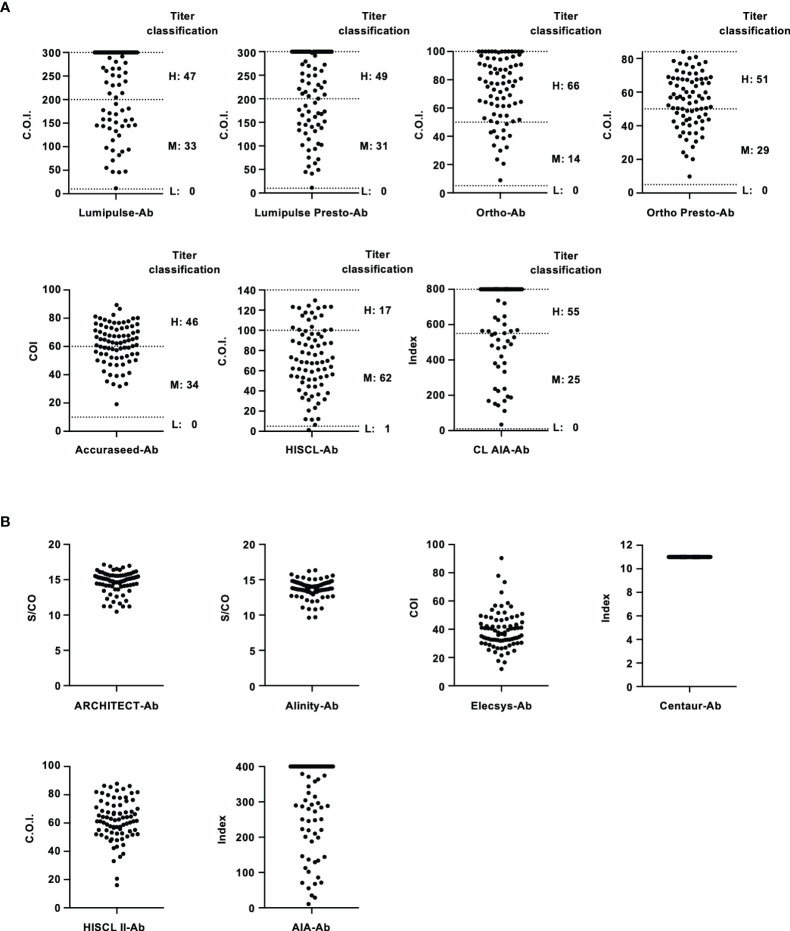
Evaluation of HCV-positive specimens in the regional reference panel by anti-HCV detection kits. **(A)** Scatter dot plot of quantified values by 7 anti-HCV detection kits with titer classification. The numbers of specimens classified as high (H), middle (M), and low (L) titers are shown on the right of each plot. Dotted lines indicate the upper detection limit and boundaries between H and M and between M and L. **(B)** Scatter dot plot of quantified values by 6 anti-HCV detection kits without titer classification.

The thirteen anti-HCV detection kits evaluated in this study are listed in **Tables 1** and **2**. Of these anti-HCV detection kits, seven kits were capable of the classifying the titers as high, middle, and low (**Table 1**), and six kits did not provide this titer classification but diagnosed specimens as positive, pending, or negative (**Table 2**). All the kits diagnosed all the HCV-positive specimens as positive and all the HCV-negative specimens as negative in the regional reference panel. The distributions of quantified titers of HCV-positive specimens by these kits varied (**Figure 1**). Among the kits providing titer classification, the titers measured by several kits were biased toward the upper detection limits, and those by others were widely distributed within the detection range. All specimens, except for one measured by HISCL-Ab, were classified as high or middle of the titer classification, and over half of the specimens were classified as high in measurement with most kits. There were more specimens classified as middle than high by HISCL-Ab (**Figure 1A**). The distributions of anti-HCV values also varied among the kits that did not provide titer classification (**Figure 1B**).

**Table 1:** Anti-HCV detection kits providing antibody titer classification.

Kit	Principle	Manufacturer	Unit	Range	Classification			Antigen
					Low	Middle	High	
Lumipulse-Ab	CLEIA	Fujirebio	C.O.I.	1.0 - 300.0	1.0 - <10.0	10.0 - <200.0	≥200.0	C50D, NS3F, NS5aF
Lumipulse Presto-Ab	CLEIA	Fujirebio	C.O.I.	1.0 - 300.0	1.0 - <10.0	10.0 - <200.0	≥200.0	C50D, NS3F, NS5aF
Ortho-Ab	CLEIA	Fujirebio	C.O.I.	1.0 - 100.0	1.0 - <5.0	5.0 - <50.0	≥50.0	c25 (core and NS34), c33c (NS3), NS5
Ortho Presto-Ab	CLEIA	Fujirebio	C.O.I.	1.0 - 84.0	1.0 - <5.0	5.0 - <50.0	≥50.0	c25 (core and NS34), c22-3 (core), NS5
Accuraseed-Ab	CLEIA	Sanyo Chemical Industries	COI	1.00 -	1.0 - <10.00	10.0 - <60.00	≥60.00	core, NS3, NS4, NS5
HISCL-Ab	CLEIA	Sysmex	C.O.I.	1.0 - 140.0	1.0 - <5.0	5.0 - <100.0	≥100.0	core (GT-1b), NS3 (GT-1b), NS4 (GT-1a, -1b, -2a)
CL AIA-Ab	CLEIA	TOSOH	Index	1.0 - 800.0	1.0 - <10.0	10.0 - <550.0	≥550.0	NA

NA, not available; CLEIA, chemiluminescent enzyme immunoassay.

**Table 2:** Anti-HCV detection kits not providing antibody titer classification.

Kit	Principle	Manufacturer	Unit	Diagnosis			Antigen
				Negative	Pending	Positive	
ARCHITECT-Ab	CLIA	Abbott Japan	S/CO	<1.00	–	≥1.00	c100-3 (NS3 and NS4), HCr43 (core and NS3)
Alinity-Ab	CLIA	Abbott Japan	S/CO	<1.00	–	≥1.00	c100-3 (NS3 and NS4), HCr43 (core and NS3)
Elecsys-Ab	ECLIA	Roche Diagnostics	COI	<0.9	0.9 - <1.0	≥1.0	core, NS3, NS4
Centaur-Ab	CLIA	Siemens Healthcare Diagnostics	Index	<0.80	0.80 -<1.00	≥1.00	c22p (core), c200 (NS34), NS5
HISCL II-Ab	CLEIA	Sysmex	C.O.I.	<1.0	–	≥1.0	core (GT-1b), NS3 (GT-1b), NS4 (GT-1a, -1b, -2a)
AIA-Ab	IEMA	TOSOH	Index	<1.0	–	≥1.0	C50 epitope chimeric antigen (core, NS3, NS4; GT-1b and -2a)

CLIA, chemiluminescent immunoassay; ECLIA, electrochemiluminescence immunoassay; IEMA, immunoenzymometric assay.

### Evaluation of HCV RNA Quantification Kits

In this study, five HCV RNA quantification kits were evaluated (**Table 3**). Among these kits, two pairs of conventional and recently released kits provided by the same manufacturers were enrolled: ART-RNA and Alinity-RNA (Abbott Japan) and CAP/CTM-RNA2 and cobas-RNA (Roche Diagnostics). The new kit, Aptima-RNA (Hologic Japan), was also included. The dynamic ranges of these kits were comparable, and the lower limits of quantification were declared as follows: 10 IU/mL with Aptima-RNA, 12 IU/mL with ART-RNA, and Alinity-RNA, and 15 IU/mL with CAP/CTM-RNA2 and cobas-RNA. To quantify HCV RNA, Aptima-RNA uses transcription-mediated amplification (TMA), while other kits use reverse transcription-quantitative PCR (RT–qPCR).

**Table 3:** HCV RNA quantification kits evaluated in this study.

Kit	Manufacturer	Dynamic range (log IU/mL)	Target	Method
ART-RNA	Abbott Japan	1.08–8.00	5’UTR	RT–qPCR
Alinity-RNA	Abbott Japan	1.08–8.00	5’UTR	RT–qPCR
CAP/CTM-RNA2	Roche Diagnostics	1.18–8.00	5’UTR	RT–qPCR
cobas-RNA	Roche Diagnostics	1.18–8.00	5’UTR	RT–qPCR
Aptima-RNA	Hologic Japan	1.00–8.00	5’UTR	TMA

5’UTR, 5’ untranslated region; NA, not available; RT–qPCR, reverse transcription-quantitative PCR; TMA, transcription-mediated amplification.

**Figure 2:**
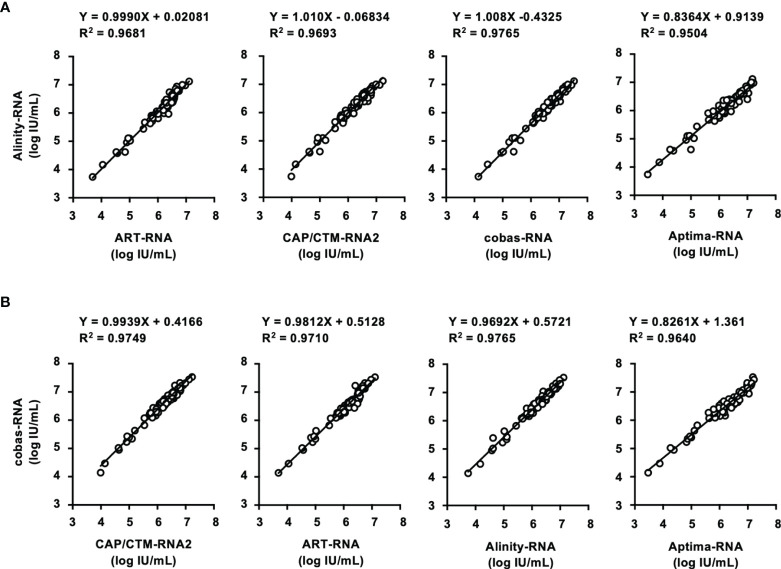
Evaluation of HCV-positive specimens in the regional reference panel by HCV RNA quantification kits. **(A)** Correlation of quantified HCV RNA titers between Alinity-RNA and ART-RNA, CAP/CTM-RNA2, cobas-RNA, and Aptima-RNA. The correlation equation and the R^2^ value are indicated at the top of the panel. **(B)** Correlation of quantified HCV RNA titers between cobas-RNA and CAP/CTM-RNA2, ART-RNA, Alinity-RNA, and Aptima-RNA. The correlation equation and the R^2^ value are indicated at the top of the panel.

To evaluate the performance of these kits, IS-RNA was serially diluted, quantified, and assessed for deviation from the theoretical titers. The quantified HCV RNA titers by these kits were close to the theoretical titer of each IS-RNA in the range of 4.0–1.5 log IU/mL, and the deviations from the assigned HCV RNA titers were < 10.0% except for one condition: 1.5 log IU/mL with cobas-RNA (**Table 4**). These kits could detect HCV RNA at a concentration of 1.0 log IU/mL, which was equal to or below the stated lower limit of quantification of these kits. In the evaluation of the regional reference panels, all these kits correctly diagnosed all specimens. The HCV RNA titers of HCV-positive specimens were distributed between 3 and 8 log IU/mL and were within the dynamic range of these kits. The quantified values by Alinity-RNA and ART-RNA, which are from one manufacturer (Abbott Japan), indicated an excellent correlation; the regression coefficient was 0.9990, and the R^2^ value was 0.9681 (**Figure 2A**). The quantified values by Alinity-RNA also showed a good correlation to the quantified values by CAP/CTM-RNA2 and cobas-RNA, which are from another manufacturer (Roche Diagnostics); the regression coefficients were 1.008 and 1.010, and the R^2^ values were 0.9765 and 0.9693, respectively. The regression coefficient between Alinity-RNA and Aptima-RNA (Hologic Japan) was lower than 0.9, although the R^2^ value was over 0.9 (**Figure 2A**). Similar correlations were observed when using cobas-RNA as a reference, and a low regression coefficient with Aptima-RNA (0.8261) was also detected (**Figure 2B**). To assess the involvement of HCV genotypes in the low regression coefficient between Aptima-RNA and other kits, we calculated the regression coefficients with each genotype. In both correlations between Aptima-RNA and either Alinity-RNA or cobas-RNA, the regression coefficients for each genotype were similarly low; 0.8257, 0.8715, and 0.8500 with Alinity-RNA and 0.8478, 0.8337, and 0.8185 with cobas-RNA for GT-1b, GT-2a, and GT-2b, respectively. The R^2^ values of these correlations did not differ among these genotypes (**Supplementary Figure S1**).

**Table 4:** Detection of HCV RNA in International Standard by HCV RNA quantification kits.

IS-RNA	Theoretical titer (log IU/mL)	4.0	3.5	3.0	2.5	2.0	1.5	1.0
ART-RNA	Titer (log IU/mL)	3.89	3.33	2.94	2.39	1.95	1.54	+
	Deviation (%)	2.75	4.86	2.00	4.40	2.50	2.67	NA
Alinity-RNA	Titer (log IU/mL)	3.78	3.45	2.93	2.34	1.96	1.37	+
	Deviation (%)	5.5	1.43	2.33	6.40	2.00	8.67	NA
CAP/CTM-RNA2	Titer (log IU/mL)	3.93	3.40	2.95	2.54	1.98	1.45	+
	Deviation (%)	1.76	2.87	1.72	1.46	0.84	3.32	NA
cobas-RNA	Titer (log IU/mL)	4.07	3.52	3.03	2.57	2.1	1.77	+
	Deviation (%)	1.78	0.54	0.94	2.85	4.90	18.06	NA
Aptima-RNA	Titer (log IU/mL)	3.87	3.32	2.86	2.29	1.94	1.37	+
	Deviation (%)	3.25	5.14	4.67	8.40	3.00	8.67	NA

+, detected but not quantifiable; NA, not available.

### Evaluation of HCV Ag Quantification Kits

**Figure 3:**
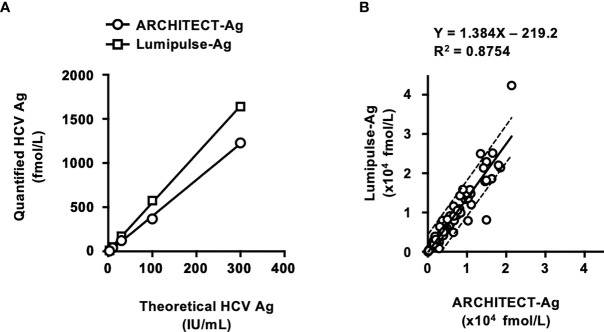
Evaluation of HCV Ag quantification kits using International Standards and a regional reference panel. **(A)** Quantified HCV Ag titers of serially diluted WHO International Standards. The WHO International Standard for HCV Ag (IS-Ag) was serially diluted to 300, 100, 30, 10, 3, and 1 IU/mL and quantified by ARCHITECT-Ag (circles) and Lumipulse-Ag (squares). **(B)** Correlations of HCV core Ag titers of the specimens in the HCV reference panel quantified by ARCHITECT-Ag and Lumipulse-Ag. Dashed lines indicate the 95% confidence intervals for the regression line. The correlation equation and the R^2^ value are indicated at the top of the panel.

In this study, two HCV Ag quantification kits, ARCHITECT-Ag and Lumipulse-Ag, were evaluated (**Table 5**). The lower limits of quantification of ARCHITECT-Ag and Lumipulse-Ag were 3.0 and 50.0 fmol/L, respectively. IS-Ag was diluted to 300, 100, 30, 10, 3, and 1 IU/mL and subjected to measurement by these kits, although these kits do not use IS-Ag as a calibrator, and the quantified data were expressed in fmol/L. ARCHITECT-Ag could measure the diluted IS-Ag to 3 IU/mL and Lumipulse-Ag could detect to 30 IU/mL, and the linearity of quantified values of the diluted IS-Ag was confirmed in both kits. However, the quantified values by these kits were 1.33–1.57-fold different from each other, indicating that these kits are not standardized (**Figure 3A**).

**Table 5:** Detection of HCV Ag in regional reference panels by HCV Ag quantification kits.

Kit	Principle	Manufacturer	Dynamic range (fmol/L)	Negative specimens diagnosed as negative (n = 70)	Positive specimens diagnosed as positive (n = 80)
ARCHITECT-Ag	CLIA	Abbott Japan	3.00–20,000.00	70 (100%)	80 (100%)
Lumipulse-Ag	CLEIA	Fujirebio	50.0–50,000.0	70 (100%)	78 (97.5%)

In the evaluation of the regional reference panel, all HCV-negative specimens were diagnosed as negative by both kits (**Table 5**). For the HCV-positive specimens, ARCHITECT-Ag quantified all 80 specimens in the HCV-positive panel, whereas Lumipulse-Ag could quantify 78 specimens (97.5%), with the HCV Ag titers of the remaining 2 specimens below the lower limit of quantification for Lumipulse-Ag. The regression coefficient of the quantified HCV Ag values by ARCHITECT-Ag and Lumipulse-Ag was 1.384, which is over 1.1, suggesting incompatibility of quantified HCV Ag data by these kits (**Figure 3B**). To assess the genotype dependency of this incompatibility, we calculated the correlations for each genotype. The regression coefficients were 1.403, 1.026, and 1.610 for GT- 1b, GT-2a, and GT-2b, respectively (**Supplementary Figure S2**). These data seemed to suggest a genotype dependence of the correlation because the regression coefficients were different in each genotype. However, when the 4 deviating specimens lying outside of the 95% confidence interval lines (C32, GT-2a; C35, GT-2b; C52, GT-2a; and C75, GT-1b) were excluded, the regression coefficients of each genotype became similar. Therefore, the correlation of the quantified values by these HCV Ag quantification kits did not seem to be affected by genotypes but was influenced by several specimens that had inconsistent values based on these two kits.

### Correlation Between Quantification of HCV RNA and HCV Ag

**Figure 4:**
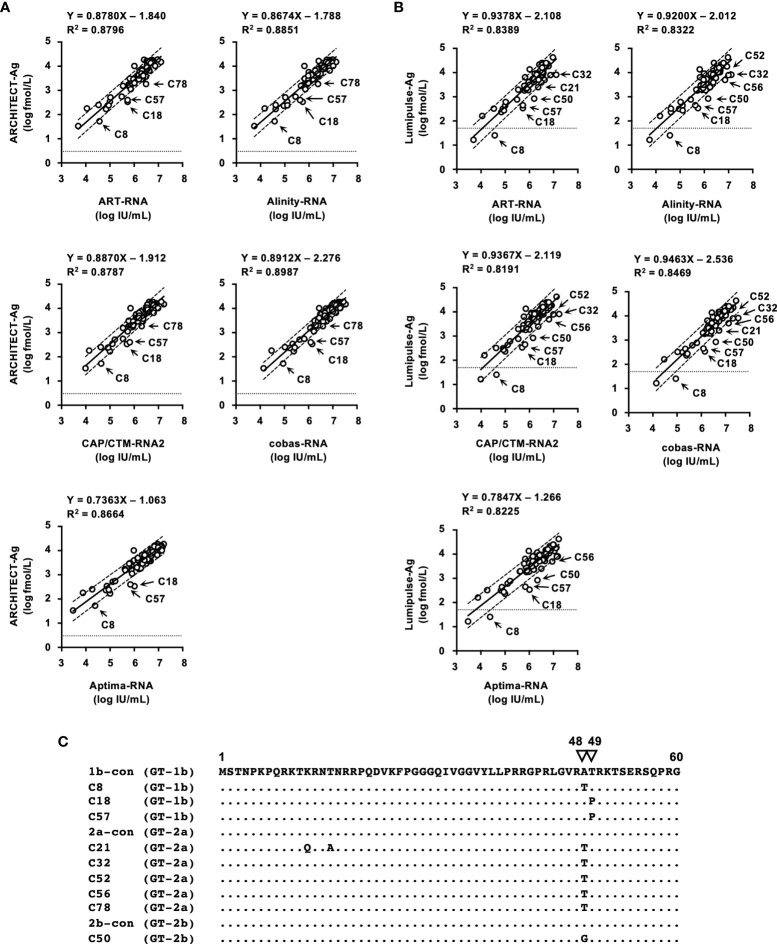
Correlations between HCV RNA and HCV Ag quantified by ARCHITECT-Ag **(A)** and Lumipulse-Ag **(B)**. Data for HCV Ag levels were converted to log fmol/L prior to analysis. In each plot, the lower limit of quantification of the respective HCV Ag assay is indicated by a dotted line. Dashed lines indicate the 95% confidence intervals for the regression line. The correlation equation and the R^2^ value are indicated at the top of the panel. **(C)** An alignment of the first 60 amino acids of the HCV core region. Estimated amino acid sequences of nine strains with polymorphisms at amino acids 48 and 49 were aligned with the consensus sequence of each genotype: 1b-con, 2a-con, and 2b-con. Genotypes of aligned strains are indicated in parentheses. The position numbers are indicated at the top of the alignment, and amino acids at positions 48 and 49 are indicated by inverted triangles. Dots indicate identical amino acids.

The correlation between HCV Ag and HCV RNA titers of specimens in the regional reference panel was also assessed. Relatively good correlations were observed between HCV RNA and log-converted HCV Ag titers. For the quantified values by ARCHITECT-Ag and HCV RNA quantification kits, the regression coefficients ranged from 0.7363–0.8912, and the R^2^ values ranged from 0.8664–0.8987 (**Figure 4A**). For the quantified values by Lumipulse-Ag and HCV RNA, the regression coefficients ranged from 0.7847–0.9463, and the R^2^ values ranged from 0.8191–0.8469 (**Figure 4B**). Reflecting the low regression coefficients between Aptima-RNA and the other HCV RNA quantification kits, quantified values by ARCHITECT-Ag and Lumipulse-Ag exhibited low regression coefficients to Aptima-RNA. In these correlations, the quantified HCV Ag values of several specimens were plotted below the lower 95% confidence interval lines. In the correlation between quantified values by ARCHITECT-Ag and HCV RNA quantification kits, 4 specimens (C8, C18, C57, and C78) were identified to be plotted below the lower 95% confidence interval lines. In the correlation between quantified values by Lumipulse-Ag and HCV RNA kits, 7 specimens (C18, C21, C32, C50, C52, C56, and C57) were identified to be plotted below the lower 95% confidence interval lines. Specimen C8 was excluded because it was under the lower limit of quantification by Lumipulse-Ag. Taken together, all these 9 specimens (C8, C18, C21, C32, C50, C52, C56, C57, and C78) were selected, and the nucleotide sequences of the HCV core region were determined. The predicted amino acid (aa) sequences were aligned with the consensus sequence of each genotype: 1b-con, 2a-con, and 2b-con (**Figure 4C**). These 9 specimens, 3 with the GT-1b strain (C8, C18, and C57), 5 with the GT-2a strain (C21, C32, C52, C56, and C78), and 1 with the GT-2b strain (C50), were shown to have polymorphisms at aa48 or aa49.

## Discussion

Postmarketing surveillance is a survey conducted after a medical device has been released to ensure its quality, efficacy, and accuracy and is used to gather detailed information that was not available before the sale. For the postmarketing surveillance of *in vitro* diagnostic kits for HCV infection, we exploited the regional reference panel for HCV consisting of epidemic strains in Japan. This reference panel was originally established to generate data regarding correlations to the preexisting kits at the approval step of a new *in vitro* screening and diagnostic kit. We also used International Standards for HCV RNA and HCV Ag for the evaluation of the performance of these kits.

Although numerous anti-HCV detection kits are currently available in Japan, we evaluated 13 representative kits in this study. All these kits correctly diagnosed all the HCV-positive and HCV-negative specimens in the reference panel. The distribution of measured titers was different among these kits, and the ratios of the titer classification were also inconsistent among the 7 kits that were available for the titer classification. The antigens used for the detection of anti-HCV in these kits differed across kits (**Tables 1** and **2**), and this difference in antigen may be responsible for the difference in anti-HCV titer. The detection of anti-HCV is used to screen for past exposure to and current infection of HCV. To distinguish between HCV carriers and persons previously infected with HCV, the anti-HCV titer was measured, and then HCV Ag or HCV RNA was detected, if necessary. In Japan, following the instructions of the Ministry of Health, Labour and Welfare of Japan, HCV screening is performed as follows: HCV antibody tests with titer classification are used to screen for current infection of HCV at the health check, and values of anti-HCV are classified into groups with high, medium, or low titers or as negative. Those with high titers or those with medium or low titers and HCV RNA positivity have a ‘high possibility of currently infected with HCV’ and are recommended to see a doctor for a definitive diagnosis and treatment. Those with medium or low titers who were HCV RNA-negative are determined to be ‘likely not currently infected with HCV’. Therefore, the titer classification of anti-HCV plays an important role in the flow of HCV screening in Japan, and the kits for this purpose are manufactured and sold only in Japan. Postmarketing surveillance of anti-HCV detection kits not only with high- or middle-titer specimens as used in this study but also with low-titer specimens will be essential to evaluate and maintain the performance of currently available or upcoming anti-HCV detection kits.

We evaluated the 5 HCV RNA quantification kits using IS-RNA and the reference panel. In the evaluation with IS-RNA, all these kits quantified the serially diluted IS-RNA down to the concentration of the quantification limits of these kits. The deviations in the quantified data by these kits from assigned concentrations were acceptable. The correlations between the quantified data by these kits was also evaluated by using the reference panel. Most of the kits exhibited excellent correlations, and the regression coefficients and the R^2^ values were within the requirements of the regulatory authority. Only the regression coefficients between the quantified values by Aptima-RNA and the other kits were lower than 0.9, although the R^2^ values were over 0.9 and genotype dependency was not detected. These data indicated that the data quantified by Aptima-RNA were not always identical to the data quantified by the other kits. The differences in the quantified data by Aptima-RNA and other kits were a maximum of 0.76 log IU/mL. Similar to our observation, the difference in the quantified data by Aptima-RNA and other kits has been previously reported, whereas an excellent correlation between Aptima-RNA and other kits indicated with a regression coefficient close to 1.0 has also been reported (Schalasta et al., 2016; Chevaliez et al., 2017; Schonning et al., 2017; Worlock et al., 2017; Chevaliez et al., 2020). Because the data quantifying the International Standards by each kit were nearly identical in each dilution, this discrepancy may depend on the region-specific genotypes or specific mutations of evaluated specimens; the evaluation of specimens obtained in a specific area, such as those included in the regional reference panel, will be essential to obtain valid data on the correlation between two kits. At least in Japan, careful attention will be needed when the HCV RNA quantification kit is changed from Aptima-RNA to another kit or another kit to Aptima-RNA during the follow-up of patients with chronic hepatitis C.

The performance of 2 HCV Ag quantification kits, ARCHITECT-Ag and Lumipulse-Ag, was also investigated. To standardize HCV Ag kits, the first WHO International Standard for HCV Ag was issued in 2014. However, it has not yet been commonly used and has not been applied to these kits. We used IS-Ag and the reference panel to evaluate the performance of these kits. As expected, the quantified data from the serially diluted IS-Ag were different from each other, and the regression coefficient in the correlation of the quantified HCV Ag values of the reference panel was over 1.1. These data suggested the incompatibility of quantified data by these kits. This incompatibility may cause problems in the clinical use of these kits because HCV Ag quantification is used as an alternative to HCV RNA to diagnose HCV infection and the evaluation of the pathological condition or therapeutic effects of chronic hepatitis C (Tanaka et al., 2003; Wang et al., 2021). In addition, the underestimation of HCV Ag values based on the correlation with HCV RNA has also been reported in some specimens. The amino acid polymorphisms at aa47–aa49 in the HCV core region were responsible for this deviation (Saeed et al., 2009; Murayama et al., 2012; Momose et al., 2018). In this study, 9 specimens in the reference panel exhibited underestimated values of HCV Ag and were revealed to have amino acid polymorphisms at aa48 and aa49. The affected specimens varied by kits; 4 specimens were underestimated when measuring with ARCHITECT-Ag, and 7 specimens were underestimated with Lumipulse-Ag (1 specimen was out of the quantification range). Specimen C78 was underestimated only by ARCHITECT-Ag, and specimens C32, C50, C52, and C56 were underestimated only by Lumipulse-Ag. The difference in these affected specimens also influenced the correlation between the quantified values by these kits. The quantified values for C32 and C52 deviated from the correlation of quantified values of these kits and fell outside of the 95% confidence interval lines. Therefore, to reconcile the differences in the quantification by these kits, standardization with IS-Ag and an investigation evaluating the influencing factors for the HCV Ag values is needed.

In this study, important information was obtained by postmarketing surveillance. The detection kits for anti-HCV and the quantification kits for HCV RNA and HCV Ag were confirmed to have enough sensitivity to diagnose HCV infection. However, the quantified values of these parameters by some kits were revealed to be incompatible with each other. In such cases, the correlation equation obtained by quantification of the reference panel will be useful for conversion. In the quantification of HCV Ag, careful attention is needed when following up the patients infected with HCV with the amino acid polymorphisms affecting the quantified values. As indicated in this study, the evaluation with International Standards and the regional reference panel was useful to confirm the quality of currently available diagnostic kits for HCV infection, and such quality control is essential for the clinical usage of these kits.

## Data Availability Statement

The datasets presented in this article are not readily available because the raw data measured by the manufacturers cannot be provided. Requests to access the datasets should be directed to TK, takato@nih.go.jp.

## Author Contributions

AM, HM, IH, and TK conceived this study. AM, HM, NY, and KM carried out the experiments. KM gathered and prepared the materials. MM and TK supervised the experiment and project. All authors contributed to the article and approved the submitted version.

## Funding

This work was performed as part of a project for the preparation of reference panels of infectious disease specimens at the National Institute of Infectious Diseases in Japan. This work was also supported by the Research on Regulatory Science of Pharmaceuticals and Medical Devices from the Japan Agency for Medical Research and Development (AMED) (JP21mk0102146). The funders played no role in the study design, data collection and interpretation, or the decision to submit the work for publication.

## Conflict of Interest

The authors declare that the research was conducted in the absence of any commercial or financial relationships that could be construed as a potential conflict of interest.

## Publisher’s Note

All claims expressed in this article are solely those of the authors and do not necessarily represent those of their affiliated organizations, or those of the publisher, the editors and the reviewers. Any product that may be evaluated in this article, or claim that may be made by its manufacturer, is not guaranteed or endorsed by the publisher.
