# Supplementary material for: Performance Evaluation of In Vitro Screening and Diagnostic Kits for Hepatitis C Virus Infection
Source: Front Cell Infect Microbiol. 2022 Feb 3;11:793472. doi: 10.3389/fcimb.2021.793472 (PMC8851425; doi:10.3389/fcimb.2021.793472)
Supplement: Supplementary file 1 [file DataSheet_1.pdf]

Supplementary Materials

Performance Evaluation of *In Vitro* Screening and Diagnostic Kits for Hepatitis C Virus Infection

Asako Murayama, Haruka Momose, Norie Yamada, Keiji Matsubayashi, Masamichi Muramatsu, Isao Hamaguchi, Takanobu Kato.

Supplementary Figure S1.

Genotype dependency of the regression coefficient for HCV RNA measured by Aptima-RNA and other kits. (A) The correlation of HCV RNA titers of the specimens in the regional reference panel by Alinity-RNA and Aptima-RNA (left) and cobas-RNA and Aptima-RNA (right). HCV GT-1b, GT-2a, and GT-2b are indicated by red, blue, and green symbols, respectively. (B) The regression coefficients and the R^2 values in each genotype are indicated.

Supplementary Figure S2.

Genotype dependency of the regression coefficient for HCV Ag quantified by Lumipulse-Ag and ARCHITECT-Ag. (A) The correlation of HCV Ag titers of the specimens in the regional reference panel. GT-1b, GT-2a, and GT-2b are indicated by red, blue, and green symbols, respectively. Specimens outside the 95% confidence intervals (dashed lines) are indicated. (B) Correlation with specimens excluding 4 deviated specimens. (C) The regression coefficients and R^2 of all specimens and each genotype are indicated.

Supplementary Figure S1

A

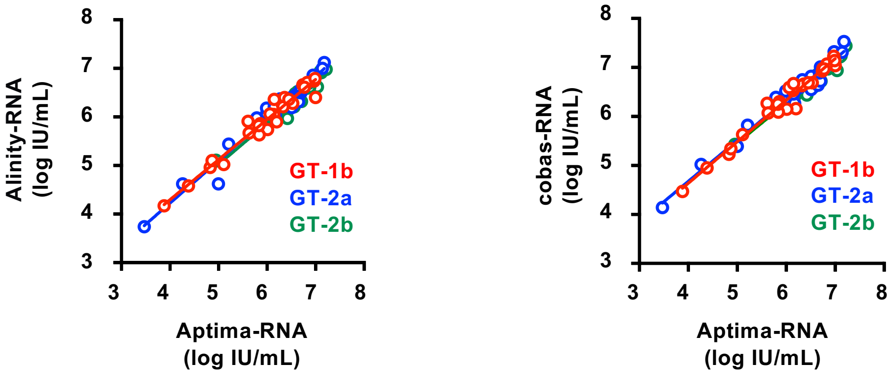

B

Compared kits	Genotype	Regression coefficient	R^2
Alinity-RNA vs. Aptima-RNA	1b	0.8257	0.9555
	2a	0.8715	0.9587
	2b	0.8500	0.9242
cobas-RNA vs. Aptima-RNA	1b	0.8478	0.9683
	2a	0.8337	0.9667
	2b	0.8185	0.9473

Supplementary Figure S2

A

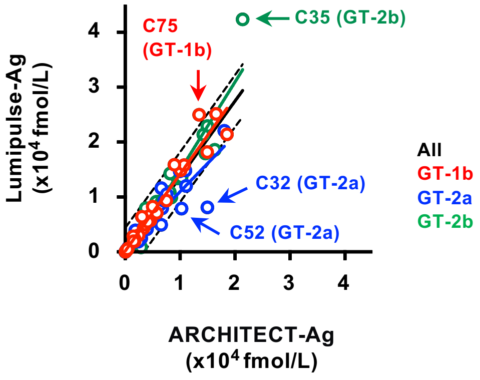

B

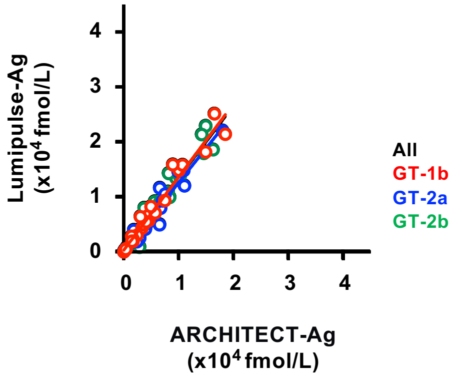

C

Compared kits	Genotype	Correlation with all specimens		Correlation with specimens excluding 4 deviated specimens	
		Regression coefficient	R^2	Regression coefficient	R^2
Lumipulse-Ag vs. ARCHITECT-Ag	All	1.384	0.8754	1.318	0.9478
	1b	1.403	0.9447	1.332	0.9615
	2a	1.026	0.7991	1.255	0.9426
	2b	1.610	0.9078	1.338	0.9355
